# Design and synthesis of tetrahydrophthalimide derivatives as inhibitors of HIV-1 reverse transcriptase

**DOI:** 10.1186/2191-2858-3-8

**Published:** 2013-08-22

**Authors:** Ashok Penta, Swastika Ganguly, Sankaran Murugesan

**Affiliations:** 1 Department of Pharmacy, Birla Institute of Technology & Science, Pilani Rajasthan 333031, India; 2 Department of Pharmaceutical Sciences, Birla Institute of Technology, Mesra-Jharkhand 835215, India

**Keywords:** NNRTIs, HAART, HIV-1 reverse transcriptase, Docking, Molecular properties, Autodock, Tetrahydrophthalimide

## Abstract

**Background:**

Non-nucleoside reverse transcriptase inhibitors (NNRTIs) are one of the key components in highly active anti-retroviral therapy because of their high specificity and less toxicity. NNRTIs inhibit reverse transcriptase enzyme by binding to the allosteric site, which is 10Å away from the active site. Rapid emergence of resistance is the major problem with all anti-HIV agents. Hence, there is continuous need to develop novel anti-HIV agents active against both drug sensitive and resistance strains.

**Results:**

All the 16 synthesized 2-(1,3-dioxo-3a,4-dihydro-1*H*-isoindol-2(3*H*,7*H*,7a*H*)-yl)-*N*-(substitutedphenyl) acetamide **4(a-p)** analogs were characterized by Fourier transform infrared spectroscopy, proton nuclear magnetic resonance spectroscopy, mass spectroscopy, and elemental analysis. Lipinski rule of five parameters and molecular parameters like solubility, drug likeness, and drug score were derived for designed analogs using online servers like Molinspiration and Osiris property explorer. Synthesized compounds were evaluated for their HIV-1 reverse transcriptase inhibitor activity by HIV-1 RNA-dependent DNA polymerase activity assay at 2 and 20 μM concentrations.

**Conclusions:**

Among the 16 synthesized compounds, **4a**, **4b**, **4f**, **4g**, **4k**, and **4l** showed weak reverse transcriptase inhibitor activity at 20 μM concentration. For the designed compounds, there was no correlation observed between molecular modeling and *in vitro* studies.

## Background

Acquired immune deficiency syndrome (AIDS) is the advance stage of infection caused by the human immunodeficiency virus (HIV-1). AIDS and AIDS-ailed infections are major leading causes of death. According to *UNAIDS*-2012 report, 33 million people are living with AIDS and 1.7 million people died in the year 2011 [1]. Highly active anti-retroviral therapy (HAART), a combination of two nucleotide or nucleoside reverse transcriptase inhibitor (NRTIs) and one protease inhibitor (PI), is generally used for AIDS. Alternative combinations like two NRTIs and one non-nucleoside reverse transcriptase inhibitor (NNRTI) or two NRTIs and one integrase inhibitor are used. NNRTIs are the key components in HAART because of their high potency, selectivity, and less toxicity when compared to NRTIs and PIs [2,3]. Currently five NNRTIs are approved by United States Food and Drug Administration. Among them, nevirapine, delavirdine, and efavirenz are first generation, which already got resistance. Etravirine and rilpivirine are potent and currently using second generation NNRTIs. However, the occurrence of the high mutation rate of the virus and the resulting emergence of resistance make the researchers run a never-ending marathon to keep developing new drugs active against both drug-sensitive and drug-resistance strain with better therapeutic profile [4,5].

Many NNRTIs, including tetrahydroimidazo [4,5,1-jkj][1,4] benzodiazepin-2(1*H*)-one and α-anilinophenyl acetamide derivatives, adapt typical butterfly-like conformations in non-nucleoside inhibitory binding pocket (NNIBP), with one hydrophilic body and two hydrophobic wings (wing-1 and wing-2). Hydrophilic body contains mainly functional groups like -NH, -C=O, and -OH which are able to form hydrogen bonding interactions with active site amino acids like K101, K103, and P236. Hydrophobic wings are *π*-electron containing aromatic ring system, which can form hydrophobic interactions and pi-cationic interactions with amino acids Y181, Y188, W229, F227, V106, P236, L100, L234, and Y318 [6,7].

**Figure 1:**
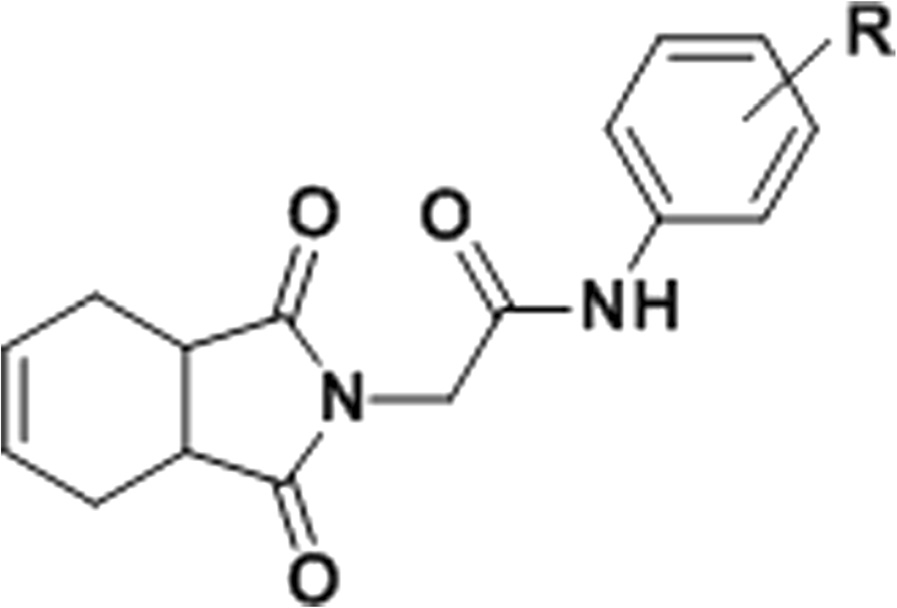
General structure of synthesized compounds.

Compounds having phthalimide scaffold exhibit anti-inflammatory [8], anticancer [9], antibacterial [10], HIV-1 RT [11,12], and HIV-1 integrase inhibitory [13] activities. An extensive perusal of literature revealed that little work has been done on phthalimides and tetrahydrophthalimide as NNRTIs. In view of these facts and our interest on the development of novel NNRTIs, we have chosen tetrahydrophthalimide scaffold as one of the hydrophobic wings in butterfly-shape pharmacophore. All the newly synthesized compounds were designed based on the derived pharmacophoric model with acetamide moiety as hydrophilic body, and tetrahydrophthalimide and substituted aromatic amines as hydrophobic wings (general structure shown in Figure 1).

## Methods

### Molecular docking study

**Figure 2:**
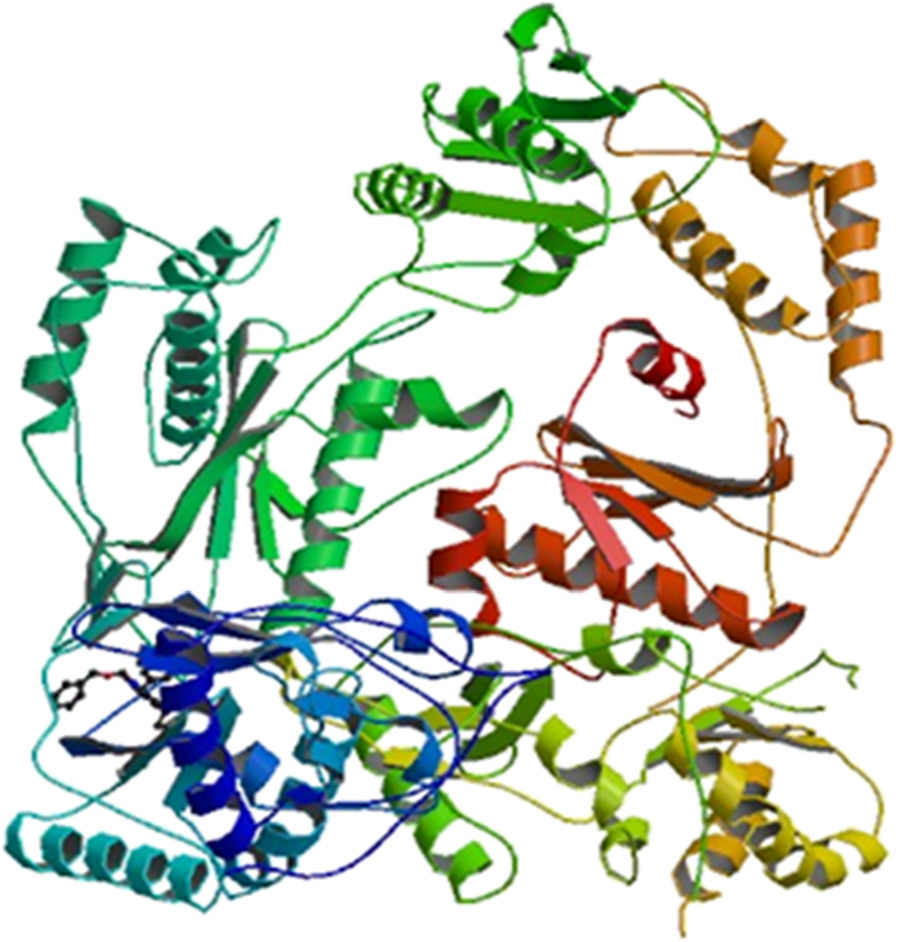
**Structure of HIV-1 reverse transcriptase co-crystallized with TNK-651**[14]**.**

The docking studies of all the derivatives **4(a-p)** were performed using molecular modeling software Autodock 4.2 (The Scripps Research Institute, CA, USA) [14] installed on a single machine running on a 3.4-GHz Pentium processor with Windows XP SP2 as the operating system. HIV-1 RT enzyme (pdb code 1rt2 (shown in Figure 2)) was taken from the RCSB, used as target protein [7]. Target protein pdb was further refined by removal of water molecules and by adding polar hydrogens and Kollmancharges. For the docking, a grid spacing of 0.375 Å and 63 × 63 × 63 number of points were used. The grid was centered on the active site. The auto grid program generated separate grid maps for all atom types of the ligand structures and one for electrostatic interactions. PRODRG online server was used to generate the energy minimized conformations of the ligands in pdb format [15]. Energy minimized conformation of ligands was subjected to calculation of Gasteiger-Huckel charges and saved in default format of Autodock. Autodock generated 50 possible binding conformations, i.e., 50 runs for each docking by using LGA search. Default protocol was applied, with initial population of 150 randomly placed individuals, a maximum number of 2.5 × 10^5^ energy evaluations and 2.7 × 10^4^ generations. A mutation rate of 0.02 and a crossover rate of 0.8 were used.

### Validation of docking

**Figure 3:**
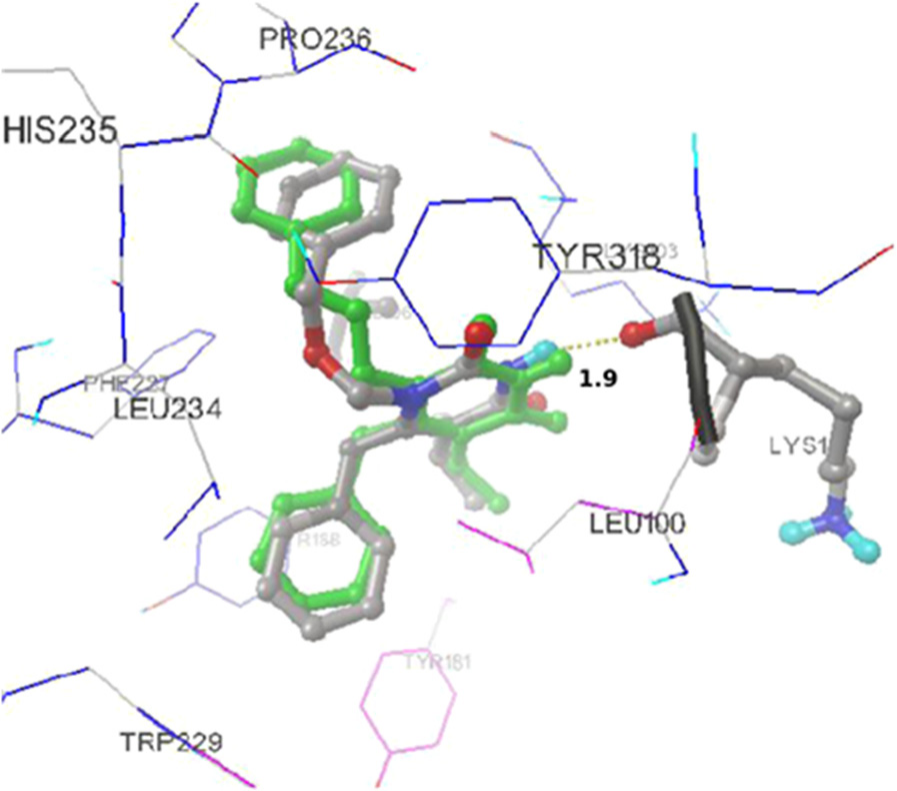
**Redocked mode of TNK 651 (3) (green) superimposed with the co-crystallized ligand (gray).** Ligand is shown as stick model, and the amino acid residues interacting with the ligands are shown as line model. Hydrogen bond interaction (1.9 Å) with LYS 103 amino acid residue of reverse transcriptase is shown as dotted spheres. The rest of the protein is suppressed for clarification purposes.

**Figure 4:**
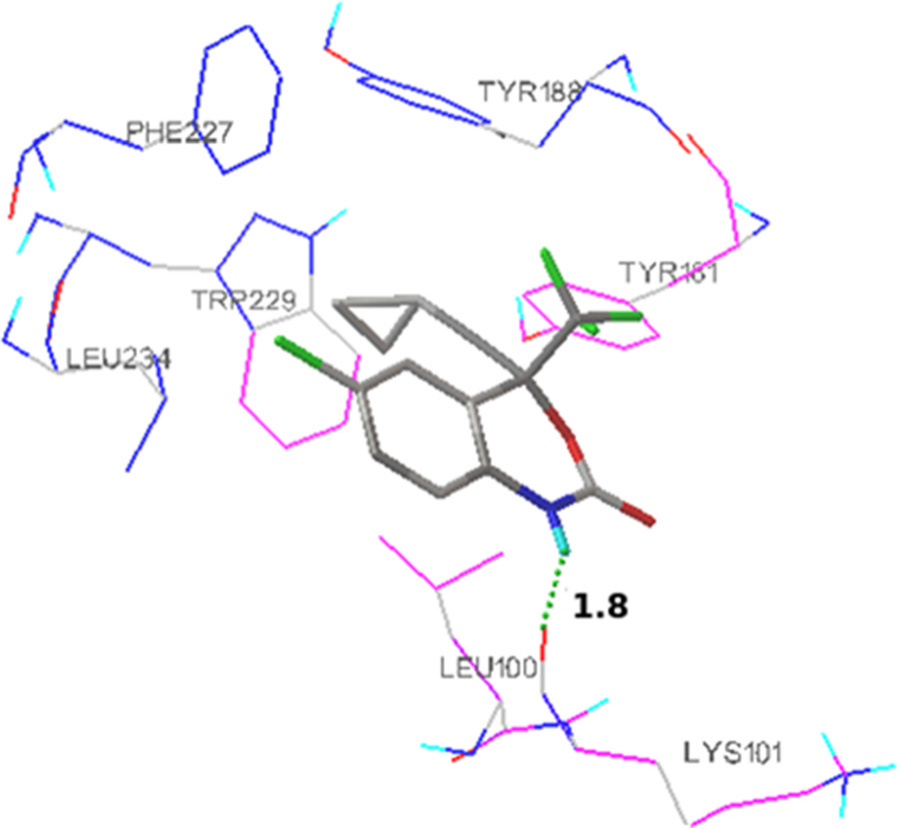
**Binding mode of standard drug efavirenz in the NNIBP of HIV-1 RT (1rt2).** Ligand is shown as stick model, and the amino acid residues interacting with the ligands are shown as line model. Hydrogen bond interactions (1.8 Å) with LYS 101 amino acid residues of reverse transcriptase respectively are shown as dotted spheres. The rest of the protein is suppressed for clarification purposes.

Initially, the receptor was docked with extracted ligand TNK 651 in order to validate the docking calculations, reliability, and reproducibility of the docking parameters for the study. It was evident that the docked pose of the re-docked ligand was almost superimposed with that of the co-crystallized ligand (Figure 3) with RMSD value of 0.5. Then, docking was performed with the standard drug efavirenz with 1tr2 for validation, and the mode of interaction was shown in Figure 4.

**Figure 5:**
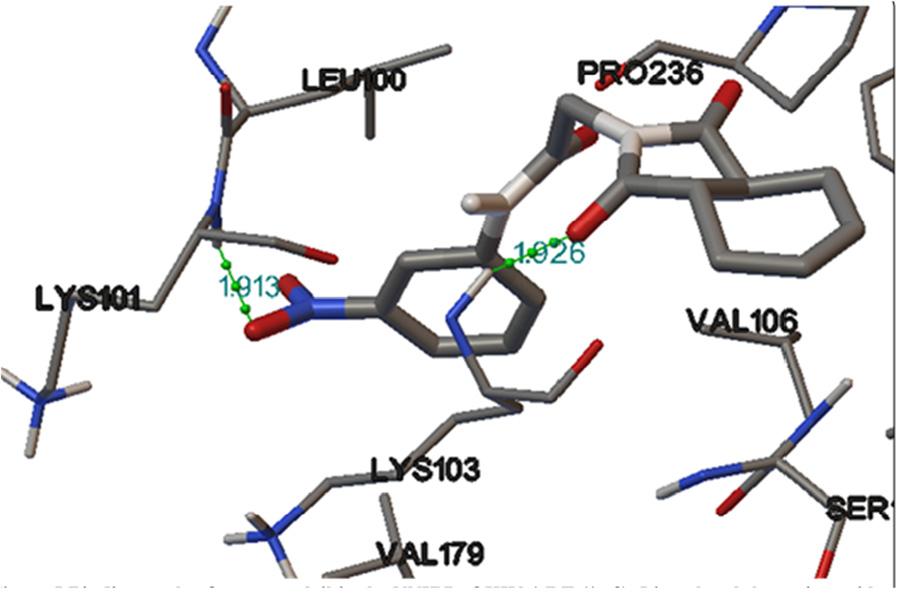
**Binding mode of compound 4l in the NNIBP of HIV-1 RT (1rt2).** Ligand and the amino acid residues interacting with the ligands are shown as ball-and-sticks model. Hydrogen bond interactions (1.913 Å) with LYS 101 and (1.926 Å) with LYS 103 amino acid residues of reverse transcriptase are shown as dotted spheres. The rest of the protein is suppressed for clarification purposes.

**Figure 6:**
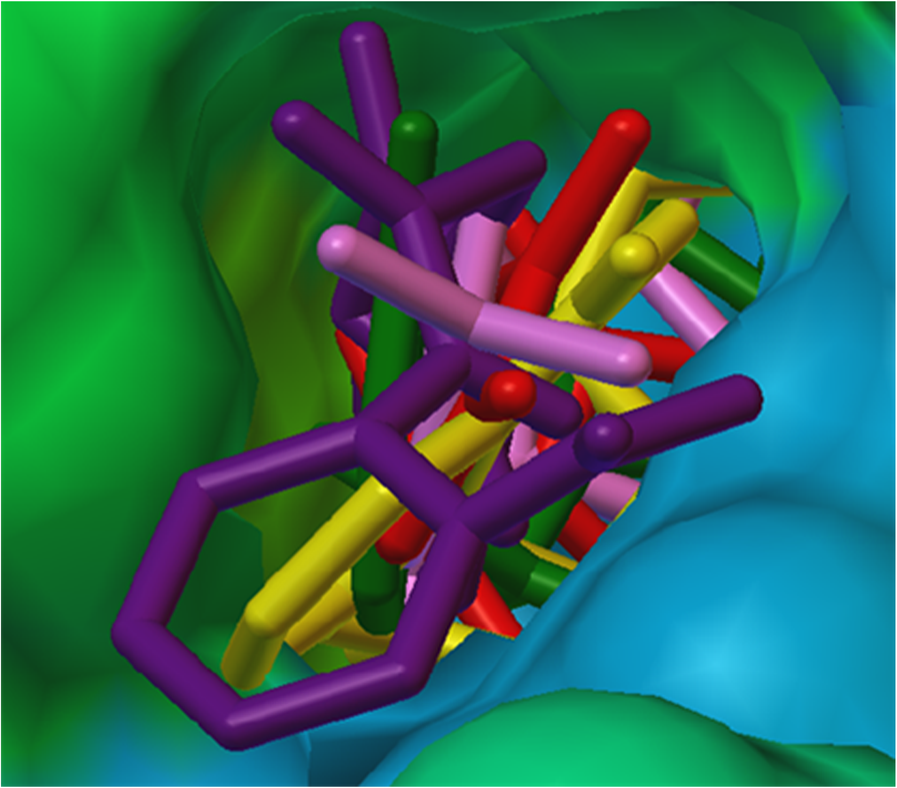
**Overlay stereoview.** 4k (pink), 4l (yellow), 4m (violet), 4o (red), and 4p (green)in the NNIBP of HIV-1 RT.

The binding free energies (docking score) and predicted inhibitory constant (Ki) values of the designed analogs were compared with binding free energies and inhibitory constants of the co-crystallized ligand TNK-651 and standard drug efavirenz. Binding free energies and predicted inhibitory constant values of TNK-651, efavirenz, and designed analogs were given in Table 1. Docking studies of designed compounds have shown satisfactory results. Hydrophilic body of designed analogs showed hydrogen bonding interactions with amino acids of receptor protein 1rt2. Hydrogen bonding interactions of compound 4l with LYS 101 and LYS 103 are shown in Figure 5. All the designed analogs showed similar orientation in NNIBP of receptor protein. The orientation of some designed compounds (having low binding free energy) in NNIBP of receptor is shown in Figure 6.

**Table 1:** Binding free energy and predicted inhibitory constant values of the synthesized compounds

**Serial**	**Compound**	***R***	**Binding**	**Inhibitory**
**number**	**code**		**free energy**	**constant (nM)**
			**(Kcal/mole)**	
1	Efavirenz	-	−12.02	1.56
	(standard)			
2	TNK-651	-	−11.88	1.95
3	4a	H	−8.13	1,100.0
4	4b	4-OCH_3_	−7.65	2,460.0
5	4c	4-CH_3_	−8.5	588.3
6	4d	4-Cl	−7.79	1,930.0
7	4e	3-OCH_3_	−8.62	479.1
8	4f	3-CH_3_	−8.61	484.7
9	4g	3-Cl	−8.87	315.7
10	4h	2-OCH_3_	−7.94	1,510.0
11	4i	2-CH_3_	−8.2	975.83
12	4j	2-Cl	−8.2	975.83
13	*4k*	*4-NO*_*2*_	*−10.53*	*19.11*
14	*4l*	*3-NO*_*2*_	*−10.89*	*10.38*
15	*4m*	*2-NO*_*2*_	*10.46*	*22.86*
16	4n	2,4-diCH_3_	−8.87	313.63
17	*4o*	*3,4-diCH*_*3*_	*10.49*	*20.37*
18	*4p*	*2-Cl, 3-CH*_*3*_	*−10.71*	*14.2*

*4k*, *4l*, *4m*, *4o*, and *4p* showed satisfactory and comparable docking results as that of standard drug efavirenz and TNK-651 (the same thing has been discussed under the subsection “*In vitro* HIV-1 RT inhibitory activity” of the “Results and discussion” section).

### Molecular parameters

Lipinski rule of five parameters like ClogP, molecular weight, number of hydrogen bond acceptors (HBA), number of hydrogen bond donors (HBD), solubility, drug likeness, and drug score were derived through online servers Molinspiration (Molinspiration Cheminformatics, Nova Ulica, Slovak Republic) and OSIRIS (Organic Chemistry, Switzerland) property calculator [16,17]. All the calculated values were given in Table 2.

**Table 2:** Predicted molecular parameters of the synthesized compounds

**Compound code**	**CLogP**	**Molecular weight**	**Number of HBA**	**Number of HBD**	**Solubility**	**Drug likeness**	**Drug score**
4a	1.10	284	5	1	−2.45	2.29	0.7
4b	1.00	314	6	1	−2.47	2.67	0.34
4c	1.42	298	5	1	−2.79	2.54	0.42
4d	1.72	318	5	1	−3.19	4.71	0.69
4e	1.00	314	6	1	−2.47	3.65	0.43
4f	1.42	298	5	1	−2.79	3.56	0.71
4g	1.72	318	5	1	−3.19	3.93	0.69
4h	1.00	314	6	1	−2.47	4.05	0.72
4i	1.42	298	5	1	−2.79	4.07	0.71
4j	1.72	318	5	1	−3.19	4.17	0.55
4k	0.97	329	8	1	−2.91	−11.4	0.28
4l	0.97	329	8	1	−2.91	−1.34	0.27
4m	0.97	329	8	1	−2.91	−3.08	0.22
4n	1.74	312	5	1	−3.14	0.76	0.47
4o	1.74	312	5	1	−3.14	−0.22	0.32
4p	2.03	332	5	1	−3.53	3.93	0.66

## Results and discussion

### Chemistry

**Scheme 1:**
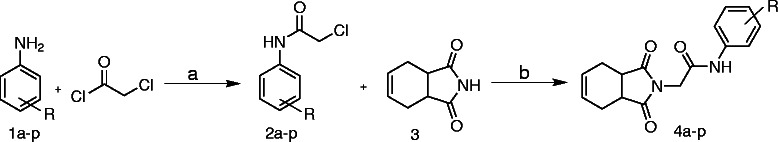
**Designed analogs synthesized using a synthetic protocol. (a)** Triethylamine, dichloromethane, room temperature, 30 min; **(b)** K_2_CO_3_, acetonitrile, reflux, 7 to 8 h.

Designed analogs were synthesized using a synthetic protocol shown in Scheme 1. In the first step, 2-chloro-*N*-(substituted phenyl) acetamide (**2a-p**) analogs were synthesized by treating substituted anilines with 2-chloroacetyl chloride in dichloromethane and triethylamine as base. 2-chloro-*N*-(substituted phenyl) acetamide (**2a-p**) intermediates were then treated with tetrahydrophthalimide (**3**) in presence of base potassium carbonate and acetonitrile as solvent to yield titled compounds as final products **4(a-p**) [18,19].

Synthesized compounds were isolated as pure and characterized by IR, ^1^H NMR, mass, and elemental analysis data. In general, the IR spectra of the synthesized compounds showed N-H stretching at around 3,408 to 3,259 cm^−1^, C = O (amide) absorption band at around 1,703 to 1,682 cm^−1^, C = O (phthalimide) absorption band at around 1,786 to 1,768 and 1,712 to 1,702 cm^−1^, C-O-C absorption (methoxy) band at around 1,249 to 1,234 cm^−1^, and C-Cl absorption band at around 697 to 678 cm^−1^. The ^1^H NMR spectrum of the product **4c** (see ‘Experimental’ section) showed two characteristic singlets at *δ* 4.27 and *δ* 2.30 because of COCH_2_-N and CH_3_, respectively. One broad singlet at *δ* 7.36 indicates the presence of NH, two doublets at *δ* 7.32, and *δ* 7.10 confirms the presence of para-substituted benzene ring. Besides these, the aliphatic region also showed the characteristic multiplet peaks due to CH = CH, CH-CH, and =CH-CH_2_ at *δ* 5.96 to 5.97, *δ* 3.21 to 3.23,*δ* 2.63 to 2.69, and *δ* 2.26 to 2.28, respectively. Mass spectral analysis of the compounds**4a** and **4c** showing the molecular ion peak at 285.6 and 299.6 (*M* + 1), respectively, confirms the molecular weight of the desired compounds.

### *In vitro* HIV-1 RT inhibitory activity

All the synthesized compounds **4**(**a-p**) were evaluated for HIV-1 RT inhibitory activity at concentrations 2 and 20 μM by using HIV-1 RT RNA-dependent DNA polymerase activity assay [20]. HIV-1 RT inhibitory activity results are shown in Table 3. Rilpivirine was used as standard drug in the assay.

**Table 3:** HIV-1 RT inhibitory activity of synthesized compounds

**Serial number**	**Compound code**	**%RT inhibition**	
		**2 μM**	**20 μM**
1	4a	NA	25
2	4b	NA	10
3	4c	NA	NA
4	4d	NA	NA
5	4e	NA	NA
6	4f	NA	20
7	4g	NA	15
8	4h	NA	NA
9	4i	NA	NA
10	4j	NA	NA
11	4k	NA	15
12	4l	NA	10
13	4m	NA	NA
14	4n	NA	NA
15	4o	NA	NA
16	4p	NA	NA

NA indicates not active.

Among the designed analogs, **4k**, **4l**, **4m**, **4o**, and **4p** showed satisfactory and comparable docking results such as free binding energy and predicted inhibitory constant (Ki) as that of standard drug efavirenz and TNK-651. Docking results encourage us towards their synthesis and *in vitro* RT inhibition evaluation. *In vitro* evaluation of these compounds (**4a**, **4b**, **4f**, **4g**, **4k**, and **4l**) showed weak HIV-1 RT inhibitory activity at 20 μM concentration. In this series of compounds **4a** (2-(1,3-dioxo-3a,4-dihydro-*1H*-isoindol-2(3*H*,7*H*,7aH)-yl)-*N*-phenylacetamide) having un-substituted phenyl ring (mentioned as wing 2 in pharmacophore) showed 25% inhibition of HIV-1 RT at tested concentration of 20 μM. Compound **4f** (2-(1,3-dioxo-3a,4-dihydro-1H-isoindol-2(3*H*,7*H*,7a*H*)-yl)-*N*-m-tolylacetamide), having m-tolyl (3-methylphenyl) group as wing 2, inhibited 20% of HIV-1 RT at 20 μM concentration. However, none of these compounds showed HIV-1 RT inhibition at 2 μM concentration.

## Experimental

All solvents and reagents purchased from Sigma (Bangalore, India) or Merck (NJ, USA) companies are used as received without further purification. Solvent system used throughout experimental work for running thin layer chromatography was ethyl acetate and hexane mixture (30:70) in order to monitor the reaction.

Melting points are uncorrected and were determined in open capillary tubes on a Precision Buchi B530 (Flawil, Switzerland) melting point apparatus containing silicon oil. IR spectra were recorded using a Jasco FTIR spectrophotometer (JASCO, Inc., USA). ^1^H NMR spectra were recorded on a Bruker DPX-400 spectrometer (Bruker India Scientific Pvt. Ltd., Mumbai) using TMS as an internal standard (chemical shifts in *δ*). The ESMS were recorded on MICROMASS Quattro-II LCMS system (Waters Corporation, Milford, USA). Elemental analysis was performed on Vario EL III M/s Elementar C, H, N, and S analyzer (Elementar Analysensysteme GmbH, Germany).

### General procedure for synthesis of the compounds

#### 3-(1,3-dioxo-3a,4-dihydro-1H-isoindol-2(3H,7H,7aH)-yl)-N-(substituted phenyl) acetamides 4

To a solution of 3a,4,7,7a-tetrahydro-1*H*-isoindole-1,3(2*H*)-dione (**3**) (2 mmol) in acetonitrile, potassium carbonate (6 mmol) and corresponding 2-chloro-*N*-(substituted phenyl) acetamides **2(a-p)** (2 mmol) were added and refluxed for 8 h. On completion of the reaction as monitored by TLC, the contents were poured on crushed ice. Resulted precipitate was filtered, dried, and recrystallized from ethanol to obtain pure product **4**.

#### 2-(1,3-dioxo-3a,4-dihydro-1H-isoindol-2(3H,7H,7aH)-yl)-N-phenylacetamide (4a)

White solid (yield 84%, MP = 96°C to 98°C). IR (KBr, cm^−1^): 3,271 (N-H), 1,776, and 1,712 (C = O, isoindole), 1,693 (C = O, amide). MS (ES^+^): *m*/*z* = 285.6 [*M* + 1]. Analytically calculated for C_16_H_16_N_2_O_3_ (%) C, 67.80; H, 5.25; N, 9.60. Found: C, 67.75; H, 5.30; N, 9.55.

#### 2-(1,3-dioxo-3a,4-dihydro-1H-isoindol-2(3H,7H,7aH)-yl)-N-(4-methoxyphenyl)acetamide (4b)

White solid (yield 92%, MP = 102°C to 104°C). IR (KBr, cm^−1^): 3,305 (N-H), 1,778, and 1,710 (C = O, isoindole), 1,697 (C = O, amide), 1,249 (C-O-C). Analytically calculated for C_17_H_18_N_2_O_4_ (%) C, 64.70; H, 5.55; N, 8.70. Found: C, 64.75; H, 5.50; N, 8.65.

#### 2-(1,3-dioxo-3a,4-dihydro-1H-isoindol-2(3H,7H,7aH)-yl)-N-p-tolylacetamide (4c)

White solid (yield 82%, MP = 100°C to 102°C). IR (KBr, cm^−1^): 3,408 (N-H), 1,772, and 1,712 (C = O, isoindole), 1,698 (C = O, amide). ^1^H NMR(400 MHz, CDCl_3_) 7.36 (brs, 1H, N*H*), 7.32 (d, *J=* 7.3 Hz, 2H, Ar*H*), 7.10 (d, *J=* 7.1 Hz, 2H, Ar*H*), 5.96 to 5.97 (m, 2H, C*H* = C*H*), 4.27 (s, 2H, C*H*_*2*_), 3.21 to 3.23 (m, 2H, C*H*-C*H*), 2.63 to 2.69 (m, 2H, C*H*H, C*H*H), 2.30 (s, 3H, C*H*_*3*_), 2.26 to 2.28 (m, 2H, CH*H*, CH*H*). MS (ES^+^): *m*/*z* = 299.6 [*M* + 1]. Analytically calculated for C_17_H_18_N_2_O_3_ (%) C, 68.60; H, 6.25; N, 9.70. Found: C, 68.65; H, 6.20; N, 9.65.

#### N-(4-chlorophenyl)-2-(1,3-dioxo-3a,4-dihydro-1H-isoindol-2(3H,7H,7aH)-yl)acetamide (4d)

White solid (yield 84%, MP = 110°C to 112°C). IR (KBr, cm^−1^): 3,363 (N-H), 1,768, and 1,706 (C = O, isoindole), 1,698 (C = O, amide), 689 (C-Cl). Analytically calculated for C_16_H_15_ClN_2_O_3_ (%) C, 60.35; H, 4.60; N, 8.85. Found: C, 60.40; H, 4.55; N, 8.90.

#### 2-(1,3-dioxo-3a,4-dihydro-1H-isoindol-2(3H,7H,7aH)-yl)-N-(3-methoxyphenyl)acetamide (4e)

White solid (yield 78%, MP = 82°C to 84°C). IR (KBr, cm^−1^): 3,259 (N-H), 1,774, and 1,712 (C = O, isoindole), 1,703 (C = O, amide), 1,234 (C-O-C). Analytically calculated for C_17_H_18_N_2_O_4_ (%) C, 64.80; H, 5.50; N, 8.65. Found: C, 64.75; H, 5.55; N, 8.70.

#### 2-(1,3-dioxo-3a,4-dihydro-1H-isoindol-2(3H,7H,7aH)-yl)-N-m-tolylacetamide (4f)

White solid (yield 76%, MP = 86°C to 88°C). IR (KBr, cm^−1^): 3,342 (N-H), 1,776, and 1,712 (C = O, isoindole), 1,682 (C = O, amide). Analytically calculated for C_17_H_18_N_2_O_3_ (%) C, 68.20; H, 6.35; N, 9.30. Found: C, 68.25; H, 6.30; N, 9.25.

#### N-(3-chlorophenyl)-2-(1,3-dioxo-3a,4-dihydro-1H-isoindol-2(3H,7H,7aH)-yl)acetamide (4g)

White solid (yield 78%, MP = 92°C to 94°C). IR (KBr, cm^−1^): 3,290 (N-H), 1,768, and 1,712 (C = O, isoindole), 1,697 (C = O, amide), 678 (C-Cl). Analytically calculated for C_16_H_15_ClN_2_O_3_ (%) C, 60.50; H, 4.20; N, 8.90. Found: C, 60.45; H, 4.15; N, 8.95.

#### 2-(1,3-dioxo-3a,4-dihydro-1H-isoindol-2(3H,7H,7aH)-yl)-N-(2-methoxyphenyl)acetamide (4h)

White solid (yield 76%, MP = 78°C to80°C). IR (KBr, cm^−1^): 3,345 (N-H), 1,774, and 1,712 (C = O, isoindole), 1,703 (C = O, amide), 1,242(C-O-C). Analytically calculated for C_17_H_18_N_2_O_4_ (%) C, 64.75; H, 5.80; N, 8.55. Found: C, 64.80; H, 5.75; N, 8.60.

#### 2-(1,3-dioxo-3a,4-dihydro-1H-isoindol-2(3H,7H,7aH)-yl)-N-o-tolylacetamide (4i)

White solid (yield 74%, MP = 92°C to 94°C). IR (KBr, cm^−1^). 3,265 (N-H), 1,772, and 1,705 (C = O, isoindole), 1,694 (C = O, amide). Analytically calculated for C_17_H_18_N_2_O_3_ (%) C, 68.55; H, 6.25; N, 9.40. Found: C, 68.50; H, 6.30; N, 9.45.

#### N-(2-chlorophenyl)-2-(1,3-dioxo-3a,4-dihydro-1H-isoindol-2(3H,7H,7aH)-yl)acetamide (4j)

White solid (yield 76%, MP = 88°C to 90°C). IR (KBr, cm^−1^): 3,302 (N-H), 1,784, and 1,702 (C = O, isoindole), 1,676 (C = O, amide), 697 (C-Cl). Analytically calculated for C_16_H_15_ClN_2_O_3_ (%) C, 60.10; H, 4.50; N, 8.95. Found: C, 60.15; H, 4.45; N, 8.90.

#### 2-(1,3-dioxo-3a,4-dihydro-1H-isoindol-2(3H,7H,7aH)-yl)-N-(4-nitrophenyl)acetamide (4k)

Yellow solid (yield 86%, MP = 125°C to 127°C). IR (KBr, cm^−1^): 3,325 (N-H), 1,779, and 1,710 (C = O, isoindole), 1,686 (C = O, amide), 1,542, 1,322 (C-NO_2_). Analytically calculated for C_16_H_15_N_3_O_5_ (%) C, 58.60; H, 4.70; N, 12.55. Found: C, 58.55; H, 4.65; N, 12.60.

#### 2-(1,3-dioxo-3a,4-dihydro-1H-isoindol-2(3H,7H,7aH)-yl)-N-(3-nitrophenyl)acetamide (4l)

Yellow solid (yield 70%, MP = 112°C to 114°C). IR (KBr, cm^−1^): 3,338 (N-H), 1,774, and 1,712 (C = O, isoindole), 1,693 (C = O, amide), 1,537, 1,327 (C-NO_2_). Analytically calculated for C_16_H_15_N_3_O_5_ (%) C, 58.45; H, 5.05; N, 12.45. Found: C, 58.50; H, 5.10; N, 12.50.

#### 2-(1,3-dioxo-3a,4-dihydro-1H-isoindol-2(3H,7H,7aH)-yl)-N-(2-nitrophenyl)acetamide (4m)

Yellow solid (yield 64%, MP = 102°C to 104°C). IR (KBr, cm^−1^): 3,331 (N-H), 1,774, and 1,714 (C = O, isoindole), 1,698 (C = O, amide), 1,531, 1,336 (C-NO_2_). Analytically calculated for C_16_H_15_N_3_O_5_ (%) C, 58.65; H, 4.80; N, 12.50. Found: C, 58.70; H, 4.85; N, 12.55.

#### N-(2,4-dimethylphenyl)-2-(1,3-dioxo-3a,4-dihydro-1H-isoindol-2(3H,7H,7aH)-yl)acetamide (4n)

White solid (yield 86%, MP = 106°C to 108°C). IR (KBr, cm^−1^): 3,284 (N-H), 1,782, and 1,712 (C = O, isoindole), 1,672 (C = O, amide). Analytically calculated for C_18_H_20_N_2_O_3_ (%) C, 69.45; H, 6.60; N, 8.75. Found: C, 69.40; H, 6.65; N, 8.70.

#### N-(3,4-dimethylphenyl)-2-(1,3-dioxo-3a,4-dihydro-1H-isoindol-2(3H,7H,7aH)-yl)acetamide (4o)

White solid (yield 80%, MP = 110°C to 112°C). IR (KBr, cm^−1^): 3,286 (N-H), 1,778, and 1,708 (C = O, isoindole), 1,697 (C = O, amide). Analytically calculated for C_18_H_20_N_2_O_3_ (%) C, 69.65; H, 6.80; N, 8.60. Found: C, 69.60; H, 6.85; N, 8.65.

#### N-(2-chloro-3-methylphenyl)-2-(1,3-dioxo-3a,4-dihydro-1H-isoindol-2(3H,7H,7aH)-yl)acetamide (4p)

White solid (yield 84%, MP = 118°C to 120°C). IR (KBr, cm^−1^): 3,356 (N-H), 1,786, and 1,714 (C = O, isoindole), 1,695 (C = O, amide), 694 (C-Cl). Analytically calculated for C_17_H_17_ClN_2_O_3_ (%) C, 61.50; H, 5.35; N, 8.65. Found: C, 61.55; H, 5.40; N, 8.70.

### HIV-1 RNA-dependent DNA polymerase activity assay

Poly(rA)/oligo(dT) was used as a template for the RNA-dependent DNA polymerase reaction by HIV-1 RT, either wild type or carrying the mutations. For the activity assay, 25 μl final reaction volume contained TDB buffer (50 mM Tris-HCl (pH 8.0), 1 mM dithiothreitol, 0.2 mg/ml bovine serum albumin, 2% glycerol), 10 mM MgCl_2_, 0.5 mg of poly(rA)/oligo(dT)10:1 (0.3 mM 3′-OH ends), and 10 mM ^3^[H]-dTTP (1 Ci/mmol), and was introduced into tubes containing aliquots of different enzyme concentrations (5 to 10 nM RT). After incubation at 37°C for indicated time, 20 μL from each reaction tube were spiked on glass fiber filters GF/C and immediately immersed in 5% ice-cold trichloroacetic acid (TCA) (AppliChem GmbH, Darmstadt). Filters were washed three times with 5% TCA and once with ethanol for 5 min, then dried, and finally added with EcoLume scintillation cocktail (ICN, Research Products Division, Costa Mesa, CA, USA) to detect the acid precipitable radioactivity by PerkinElmer Trilux MicroBeta 1450 Counter (Waltham, MA, USA).

## Conclusion

All the synthesized 3-(1,3-dioxo-3a,4-dihydro-1*H*-isoindol-2(3*H*,7*H*,7a*H*)-yl)-*N*-(substituted phenyl) acetamide **4(a-p)** analogs were evaluated for HIV-1 reverse transcriptase inhibitor activity.

Among these synthesized compounds, **4a**, **4b**, **4f**, **4g**, **4k**, and **4l** showed weak HIV-1 RT inhibitor activity at 20 μM concentration. There was no correlation observed between molecular modeling and *in vitro* studies for these synthesized compounds.

## Abbreviations

AIDS: Acquired immune deficiency syndrome; HIV: Human immunodeficiency virus; RT: Reverse transcriptase; HAART: Highly active anti-retroviral therapy; NRTI: Nucleoside reverse transcriptase inhibitor; NNRTI: Non-nucleoside reverse transcriptase inhibitor; PI: Protease inhibitor; NNIBP: Non-nucleoside inhibitory binding pocket.

## Competing interests

The authors declare that they have no competing interests.
