# Correction: Design and synthesis of tetrahydrophthalimide derivatives as inhibitors of HIV-1 reverse transcriptase

**DOI:** 10.1186/2191-2858-3-12

**Published:** 2013-12-17

**Authors:** Ashok Penta, Swastika Ganguly, Sankaran Murugesan

**Affiliations:** 1 Department of Pharmacy, Birla Institute of Technology & Science, Pilani Rajasthan 333031, India; 2 Department of Pharmaceutical Sciences, Birla Institute of Technology, Mesra-Jharkhand 835215, India

## 

It has been drawn to the publisher’s attention that this article [1] was inadvertently published with certain omissions. The authors apologize for the omissions in the published article.

The co-authors Nicolas Sluis-Cremer and Brian D. Herman, Division of Infectious Diseases, Department of Medicine, University of Pittsburgh School of Medicine, Pittsburgh, PA 15261, USA have now been added as co-authors.

The following sentence has been added to the acknowledgements section: “The research in the N.S.–C laboratory was supported by a grant from the United States of America National Institutes of Health (GM068406)”
